# Enhancing biodegradation of polycyclic aromatic hydrocarbons using bacterial consortia and biofilm

**DOI:** 10.1186/s12866-026-05144-y

**Published:** 2026-08-10

**Authors:** Mohamed Ashraf, Aly E. Abo-Amer, Mohamed Ghazy, Ahmed Labena, Ahmed Abdel-Mawgood

**Affiliations:** 1 Biotechnology Program, Institute of Basic and Applied Sciences, Egypt-Japan University of Science and Technology (E-JUST), New Borg El Arab, Alexandria, 21934 Egypt; 2 Botany and Microbiology Department, Faculty of Science, Sohag University, Sohag, 82524 Egypt; 3 Biochemistry Department, Faculty of Science, Ain Shams University, Cairo, 11566 Egypt; 4 Processes Design and Development Department, Egyptian Petroleum Research Institute (EPRI), Nasr City, Cairo 11727 Egypt

**Keywords:** Polycyclic aromatic hydrocarbons (PAHs), Phenanthrene, Naphthalene, Biofilm, Bacterial consortia

## Abstract

The release of petroleum and its constituents, such as polycyclic aromatic hydrocarbons (PAHs), into the environment poses a significant global threat due to their toxic, genotoxic, mutagenic, and/or carcinogenic properties. Using indigenous PAH-degrading bacteria to decontaminate the PAHs is an eco-friendly, convenient, and non-expensive way in comparison to other traditional physical and chemical methods. In the present study, several bacteria were isolated from petroleum-contaminated soils using a culture-enrichment technique. Phenanthrene and naphthalene were used as representative examples of the PAHs and as sole carbon and energy sources for the isolated bacteria. Three isolates (A1, A3, and B8-1) were selected as the most potent phenanthrene and naphthalene degraders. These three most potent isolates were used to build up four different consortiums, and both the individual isolates and developed consortiums were induced to set up biofilms. The results showed that the highest growth and degradation rate of phenanthrene and naphthalene were achieved at 37 °C and pH 7. Individual strains had higher phenanthrene degrading capacities compared to microbial consortiums in both planktonic and biofilm forms. Additionally, the developed-biofilm displays an increase in the degradation rate of individual isolates A1, A3, and consortiums. Finally, the selected biofilm degraders were used to investigate the PAHs degradation ability in an artificially contaminated soil. The finding of this experiment demonstrate that the biofilm mode of growth could be more effective in PAHs degradation compared to planktonic cultures, thus it can be applied effectively to remediate PAHs-contaminated soils. The most potent PAHs degraders were identified using 16 S rRNA, and the phylogenetic sequence analysis of the three isolates was affiliated as B8-1(*Bacillus paramycoides*), A1(*Bacillus paranthracis*), and A3(*Bacillus licheniformis*).

## Introduction

The petroleum industry inevitably produces a large amount of both inorganic and organic pollutants in the environment during production, transportation, and refining processes [1]. Polycyclic aromatic hydrocarbons (PAHs) are the predominant aromatic pollutants and consist of two or more fused benzene rings. Environmental threats from PAH pollution are now substantial and ongoing, especially for areas close to industrial cities [2, 3]. PAHs are discharged to the environment as an industrial by-product through different polluting anthropogenic activities such as oil spills, incomplete fossil fuel combustion, urban runoff, and industrial activities. Moreover, their low solubility, prolonged persistence, recalcitrance, potential mutagenic, toxic, and carcinogenic effects on human health have attracted significant attention for decades [4–7]. Consequently, PAHs have been classified as significant pollutants by the Environmental Protection Agency (EPA) of the United States [8]. Various traditional PAH treatment techniques have been proposed, like solvent extraction, chemical oxidation, landfill, natural attenuation, etc [9]. Compared with these costly and time-consuming technologies, biodegradation is a reliable and relatively cost-effective technique, which makes it superior to physical and chemical methods of remediating the oil-contaminated soils [1, 10]. Thus, the application of microorganisms with high capabilities to degrade hydrocarbons is one of the most important practices for bioremediating hydrocarbon-contaminated environments [11–13]. Many bacterial species have the capability of adhering to distinct surfaces to develop a biofilm structure [14]. The developed bacterial biofilms serve as an efficient biotechnological approach for pollutant removal in the bioremediation process [14]. These developed bacterial biofilms display many features, such as their high tolerance and accumulation of toxic compounds even at high concentrations [15]. Moreover, the developed biofilm mode, especially in multispecies, can apply either in-situ or ex-situ bioremediation processes [16, 17]. All of these properties provided by the developed bacterial biofilms lead to highly enhanced bioremediation processes [18, 19]. Consequently, the biofilm is a suitable option for the bioremediation of hydrophobic PAHs as it mediates the adhesion of PAH to bacterial cells for subsequent degradation [20–22].

Although several studies have reported the biodegradation of PAHs by individual bacterial strains, limited information is available regarding the combined degradation potential of bacterial consortia isolated from petroleum-contaminated environments [23]. In addition, the influence of environmental factors such as pH and temperature on the biodegradation efficiency of these isolates requires further investigation. Therefore, the present study aimed to isolate and characterize PAH-degrading bacteria from hydrocarbon-contaminated soils in Egypt and to evaluate their degradation potential individually and in consortium under optimized conditions.

## Materials and methods

### Sample collection

Petroleum-contaminated soil samples were collected in sterile plastic bags from “General Petroleum Company, Ras Gharib, Egypt”. The samples were collected from the subsurface area and transported to the laboratory for further analyses under cooling conditions (~ 4 °C) to minimize microbial contamination during transportation.

### Soil microbial community enrichment and isolation of PAHs-degrading bacteria

Mineral salt medium (MSM) [24] was used in the isolation and enrichment of soil microbial community which have the capability to utilize phenanthrene and naphthalene as sole carbon sources. The broth media was prepared, and the pH value was adjusted to 7.0 ± 0.2 using 1 N HCl or 1 N NaOH before sterilization. The enrichment was initiated with 5.0 g of the contaminated soil samples in 100 mL of MSM, supplemented with either phenanthrene or naphthalene at an initial concentration of 1000 mg/l. Both phenanthrene and naphthalene were dissolved in dimethyl sulfoxide (DMSO). Enrichment experiments were conducted by incubating the cultures at 37 °C and 150 rpm for 7 days. Successive enrichment cultures were performed three times using a fresh medium with the same composition. In order to purify the soil microbial diversity that has the ability to utilize either phenanthrene or naphthalene, MSM-agar plates were used. The enriched media were serially diluted, streaked on MSM-agar plates, and incubated at 37 °C for 7 days. Individual colonies were isolated and streaked again on new MSM-agar plates to ascertain the purity of the colonies, and the resulted single colonies were cryopreserved in 50% glycerol solution at -80 °C for further studies.

### Screening of PAHs-degrading bacteria

The screening was performed by evaluating the growth of bacterial isolates in mineral salt medium (MSM) supplemented with either phenanthrene or naphthalene at a concentration of 200 mg L⁻¹ as the sole carbon source. Uninoculated MSM supplemented with the same concentration of PAHs served as the control. The inoculum was prepared by cultivating purified bacterial isolates in nutrient broth (Oxoid, UK) at 37 °C and 150 rpm overnight. The bacterial cells were then harvested by centrifugation at 4000 rpm for 10 min and washed three times with sterile 0.9% NaCl solution to remove residual nutrients. The cell suspension was adjusted to an optical density of OD₆₀₀ = 1.0, and 1 mL of this standardized inoculum was used to inoculate 50 mL MSM containing the target PAH. The cultures were then incubated under shaking conditions for screening of PAH-degrading ability.

### Qualitative assessment of PAHs-degrading bacteria

The ability of bacteria to degrade different PAHs, phenanthrene or naphthalene, was verified qualitatively using the redox potential (oxidized/reduced) with an indicator of 2, 6-dichlorophenol indophenol (DCPIP) as previously reported [19]. The result is interpreted as a colour change from blue (oxidized) to colourless (reduced) when it is added to the culture medium. Briefly, 50 mL of MSM supplemented with either phenanthrene or naphthalene and 1% (v/v) DCPIP were added to 250 mL Erlenmeyer flasks. Then, the prepared flasks were inoculated with the purified isolates and further incubated at 37 °C and 150 rpm for 7 days.

Uninoculated MSM supplemented with the same PAH concentration and DCPIP served as a negative control to ensure that any colour change observed was due to microbial activity. After incubation, the cultures were centrifuged at 4000 rpm for 10 min to remove bacterial cells. The supernatant was then measured for colour change at an optical density of 600 nm. The colour changes were monitored daily, and the experiment was performed in duplicate.

### Quantitative assessment of PAHs-degrading bacteria

Degradation of the PAHs, phenanthrene or naphthalene, was analysed using High Performance Liquid Chromatography (HPLC) (Shimadzu, Kyoto, Japan) equipped with a UV detector. Separation was carried out using a Shim-pack XR-ODS II column (100 mm × 3.0 mm). The mobile phase consisted of methanol and 0.1% formic acid, and the analysis was performed at a flow rate of 1 mL min⁻¹. The injection volume was 20 µL, and detection was carried out at 254 nm. PAHs were extracted using n-hexane (1:1 v/v), vortexed for 5 min, centrifuged, and the organic phase was collected for HPLC analysis. The analysis was conducted to determine the degradation efficiency at an initial concentration of 200 mg L⁻¹.

### Optimization of biodegradation conditions for enriched isolates

The biodegradation conditions for the enriched isolates were optimized using the One-Factor-at-a-Time approach. The tested parameters included temperature, pH, and incubation time in order to determine the conditions that maximize the biodegradation efficiency of the selected isolates.

#### Effect of pH value

The effect of the different pH values (5–9) on the biodegradation of the selected isolates at 200 mg L⁻¹of phenanthrene and naphthalene were tested. Overnight culture of the enriched isolates was added to each flask at a certain pH value. Then, the flasks were incubated at 37 °C and 150 rpm for 7 days. Consequently, the residual PAHs were extracted with an equal volume of n-hexane. The growth and the biodegradation rate at each pH values were measured, and the optimum pH value was selected for further optimization.

#### Effect of temperature

The effect of different temperatures (25, 30, 37, 40, and 50 °C) on the growth and degradation of phenanthrene and naphthalene by the selected enriched isolates was examined at the optimized pH values.

#### Development of bacterial consortium

The most efficient enriched isolates showing the highest growth and degradation rates for phenanthrene and naphthalene were selected for the development of bacterial consortia. Each isolate was initially cultured individually, and the cell density was standardized based on the optical density (OD₆₀₀). Bacterial consortia were then prepared by mixing equal volumes of the standardized cultures to obtain a final volume of 5 mL (see Table 1).

**Table 1:** Volume added from each isolate to develop bacterial consortia

Isolate Name	B8-1	A1	A3
Consortium Name			
Consortium 1	1560 µl	3458 µl	--
Consortium 2	2375 µl	--	2630 µl
Consortium 3	--	3458 µl	1560 µl
Consortium 4	1150 µl	2550 µl	1273 µl

Different combinations of the selected isolates were constructed to evaluate their potential synergistic interactions during PAH degradation. The consortia were composed as follows: Consortium 1 (B8-1 and A1), Consortium 2 (B8-1 and A3), Consortium 3 (A1 and A3), and Consortium 4 (B8-1, A1, and A3). The volumes of each isolate used for consortium preparation are presented in Table 1. These consortia were subsequently evaluated for their phenanthrene and naphthalene degradation efficiencies as well as their biofilm formation ability.

### Induction, screening, and quantification of biofilm-forming ability

Screening for biofilm`s development was done using a glass tube assay according to the method that previously reported [25] with some modifications. Briefly, fresh-grown cell cultures were diluted to OD_550_ = 0.2 in Trypticase soy broth (TSB) medium. One mL of this suspension was transferred to a glass tube containing 1 mL of TSB medium. Then the tubes were incubated at 37 °C under static conditions for 48 h. After that, liquid cultures were carefully removed from the tubes. The tubes were then rinsed once with Phosphate buffer saline (PBS). Afterwards, the biofilms formed inside the wall of the tubes were stained with 1% Crystal violet solution for 20 min at room temperature. After washing twice with distilled water to remove the free dye, the tubes with the attached cells appear as crystal violet-stained rings and considered as a positive-biofilm result.

### Comparison between biodegradation rate of individual isolates and consortia in planktonic and biofilm growth modes

Biodegradation rate of phenanthrene (PAHs model) by individual isolates and the developed consortia in both modes of growth, planktonic and biofilm, was estimated. For planktonic–mediated biodegradation, individual isolates and consortia were grown overnight and inoculated in tubes containing 7 mL MSM medium supplemented with 200 mg L⁻¹phenanthrene. The tubes were incubated at 150 rpm at 37 °C and 150 rpm for 7 days. After the incubation period, the residual phenanthrene was extracted with an equal volume of n-hexane and quantified using HPLC. For quantifying biofilm-mediated degradation of phenanthrene, both individual isolates and consortiums were grown as a biofilm on glass tubes containing 3 mL TSB medium at 37 °C at a static condition. After 48 h, free planktonic cells were carefully removed from the glass tube, and the biofilm formed inside the walls was rinsed once with sterile water. Afterwards, 7 mL of MSM medium containing 200 mg L⁻¹ phenanthrene was added to each tube. Then the tubes were tightly sealed and incubated at 37 °C without shaking for 7 days.

### Evaluating the efficiency of biofilm cells in the degradation of PAHs in artificially contaminated soils

The ability of the selected isolate-developed biofilm to degrade the PAHs in a real environment was investigated. The selected isolate-developed biofilm was applied to an artificially contaminated soil that was supplemented with phenanthrene and naphthalene, and the rate of PAHs biodegradation was evaluated.

The selected isolate-developed biofilm was developed in advance at 37 °C for 48 h under static conditions in 100 mL conical flasks containing 50 mL of TSB medium. The liquid culture containing planktonic cells was removed from the flasks, leaving the developed biofilms inside the walls. The soil samples used in this experiment were collected from a non-contaminated environment. A 5 g sample of the soil was artificially contaminated with 200 mg/g of phenanthrene or naphthalene. Then the flasks were tightly closed and incubated at 37 °C for 7 days. Each experiment was prepared with a negative control containing no biofilm cells. At the end of the incubation period, aliquots of soil samples from each flask, and the phenanthrene or naphthalene were extracted directly from the soil samples with an equal volume of hexane (1 gm soil: 10 mL hexane). The mixtures were shaken vigorously for 3 min and centrifuged at 4000 rpm for 20 min at room temperature to separate aqueous and solvent layers. Then the solvent layer was subjected to HPLC analysis.

### Molecular identification and phylogenetic studies of the most potent isolates using 16 S rRNA

#### Isolation of bacterial DNA

The enriched bacterial isolates were grown in 15 mL of Luria broth (LB) media at 37 ℃, 150 rpm for 2 days. The DNA extraction protocol was adapted as previously reported [26]. The DNA concentration and purity were measured by Thermo-Scientific Nano-Drop 2000c.

#### Polymerase Chain Reaction (PCR) of the bacterial DNA

The 16 S rRNA gene was amplified using universal primers of 27 F “5′-AGAGTTTGATCCTGGCTCAG-3” and 1494R “5’-TACGGCTACCTTGTTACGAC-3 ‘” [27]. The PCR mixture comprised:1 µl of 10 µM 27F primer, 1 µl of 10 µM 1494R primer from KODone, 1 µl of 100-folds diluted template, 25 µl of KODone master mix (TOYOBO, Japan), and 22 µl nuclease-free water. The reaction conditions were: initial denaturation at 95 ℃ for 1 min., denaturation at 95 ℃ for 15 sec, annealing, 50 ℃ for 15 sec, extension, 68 ℃ for 10 sec, for 30 cycles, and final extension, 68 ℃ for 1 min using a Senso-Quest Lab-cycler.

#### Sequencing analyses of the PCR products

The sequence analysis of the PCR products was performed by FASMAC company (https://fasmac.co.jp/en) using the 27 F and 1494R universal primers for the 16 S rRNA gene. The sequencing was performed using the Next Generation sequencing approach on an Illumina platform. The resulting sequence was examined for sequence homology with the archived 16 S rDNA sequences from GenBank at the database of the National Centre for Biotechnology Information (NCBI) (www.ncbi.nlm.nih.gov/nucleotide) employing the BLASTN query. Moreover, the Ez-Bio-Cloud database (https://www.ezbiocloud.net/identify) was used to provide similarity-based searches against the quality-controlled databases of 16 S rRNA sequences [28]. Phylogenetic analysis was performed, and phylogenetic trees were inferred by the neighbour-joining DNA distance algorithm using MEGA software [20]. The 16SrRNA gene sequences of the three most efficient isolates have been submitted to NCBI GenBank under the accession numbers PV848451, PV848453, and PV848454, respectively.

## Results and discussion

### Isolation and screening of PAHs-degrading bacteria

**Fig. 1:**
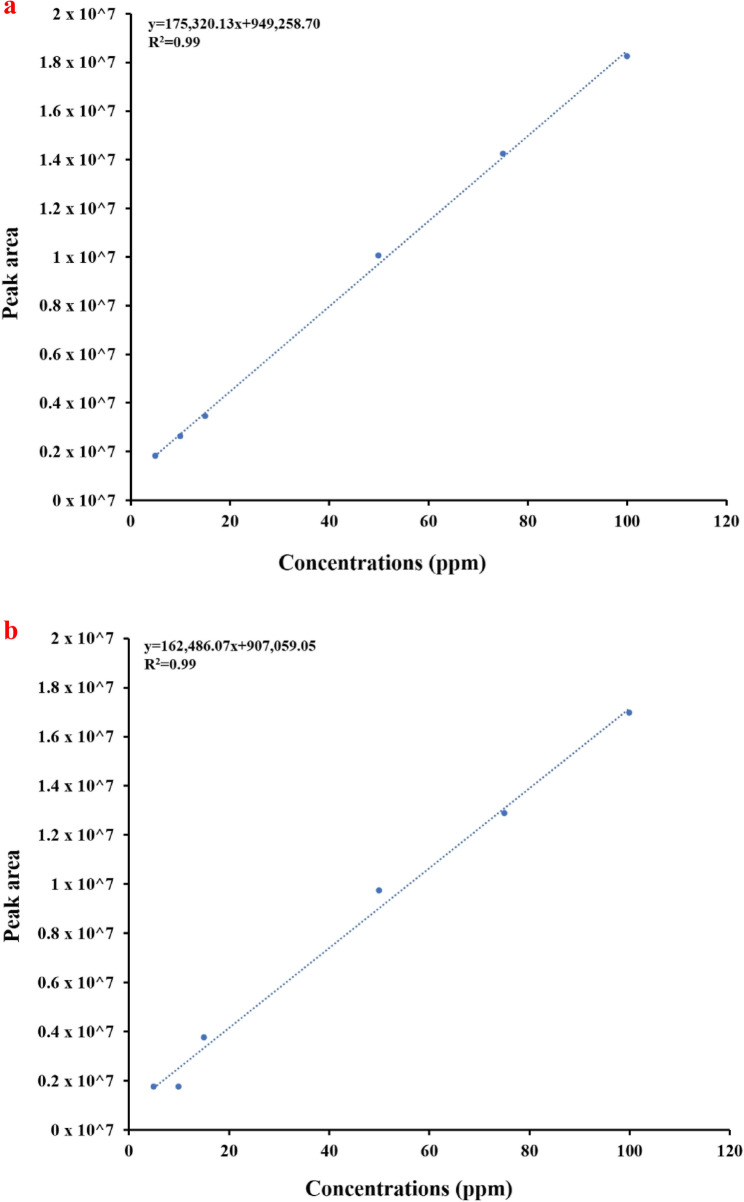
Calibration curves of (**a**) phenanthrene, and (**b**) naphthalene

After an initial enrichment of the PAHs-degrading bacteria, phenanthrene and naphthalene supplementation of soil samples, 37 colonies with different colony morphologies were isolated. The isolated bacteria were screened for their potential to utilize phenanthrene or naphthalene as a sole carbon source. The screening was evaluated by qualitatively assessing and monitoring the growth of the isolates on MSM-PAH cultures over the period of 7 days. Amongst the 37 isolates, 6 isolates (designated as B7-6, B8-1, A1, A2, A3, and A4) exhibited visible growth on MSM-PAHs-media. The bacterial isolate’s ability to degrade different hydrocarbons, phenanthrene or naphthalene, was verified using the method based on the redox indicator 2, 6-dichlorophenol indophenol (DCPIP) by observing the change in colour from blue to colourless. This is because DCPIP is an electron acceptor, which can acquire the electrons from the hydrocarbons after being degraded and transform from the oxidized form (blue) to the reduced form (colourless). (Table 2) showed that the 6 isolates were able to reduce the DCPIP using electrons from the PAHs to convert the DCPIP from blue colour, oxidized form, to colourless, reduced form. These results displayed that the isolates can utilize both phenanthrene and naphthalene as sole carbon and energy sources. Afterwards, these 6 isolates were chosen to undergo a quantitative screening, in which the concentrations of phenanthrene and naphthalene in culture media were measured by HPLC using the calibration curves formed by measuring known concentrations of phenanthrene and naphthalene (Fig. 1).

**Table 2:** Qualitative screening of the bacterial isolate’s ability to reduce DCPIP from blue to colorless

Isolate	B7-6	B8-1	A1	A2	A3	A4
Activity	+*	+	+	+	+	+

*+ cultivable on PAHs as a sole carbon source

Then degradation rate or efficiency was calculated using the equation described earlier [29] as follows:

$$\text{Degradation rate}\;(\%)\;=\;(\mathrm{C}_\mathrm{i}\;-\;\mathrm{C}_\mathrm{f}\;/\;\mathrm{C}_\mathrm{i})\;\times\;100\%$$

where C_i_ was the initial concentration of PAHs, and C_f_ was the final concentration of PAHs.

**Fig. 2:**
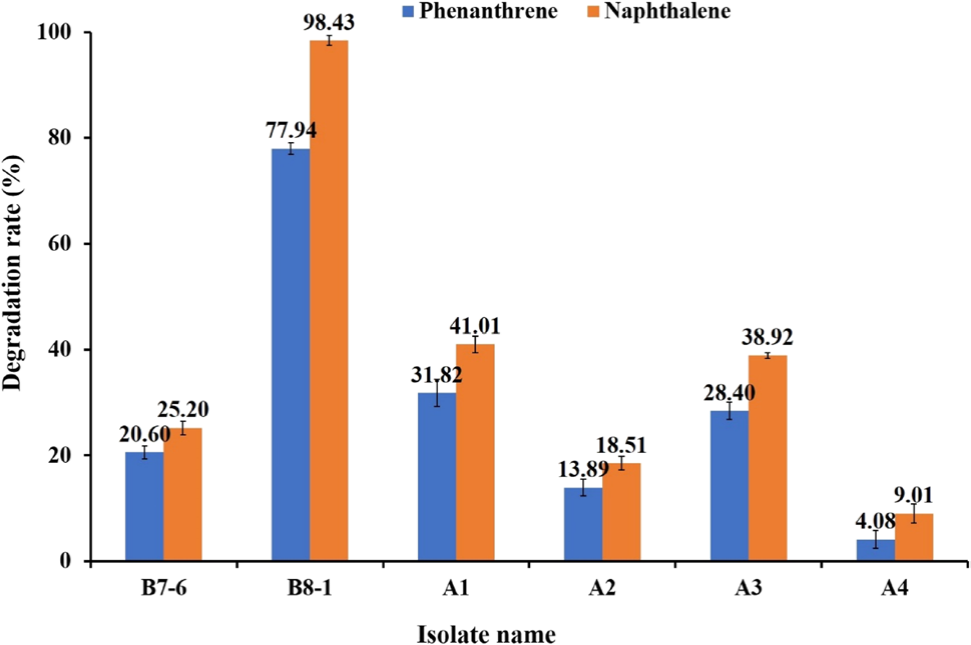
The degradation rates of phenanthrene and naphthalene using selected bacterial isolates

Among the isolated strains (B7-6, B8-1, A1, A2, A3, A4), three isolates (B8-1, A1, and A3) were selected as the most efficient PAH-degrading bacteria based on the degradation percentages determined by HPLC analysis. Among them, isolate B8-1 showed the highest degradation efficiency, with 77.94% phenanthrene degradation and 98.43% naphthalene degradation. Isolates A1 and A3 exhibited moderate degradation abilities, with phenanthrene degradation rates of 31.82% and 28.40%, respectively, and naphthalene degradation rates of 41.01% and 38.92%, respectively (Fig. 2). These isolates were therefore selected for subsequent optimization experiments.

### Optimization conditions of the biodegradation ability of bacterial isolates

The biodegradation of PAH compounds in the environment is mainly carried out through microbial processes, but several environmental factors affect the prospect of degradation of PAHs by bacteria [30]. Therefore, in the present study, the optimization conditions, effect of pH, and temperature, were evaluated to achieve the maximum biodegradation rates of phenanthrene and naphthalene.

#### Effect of pH value

**Fig. 3:**
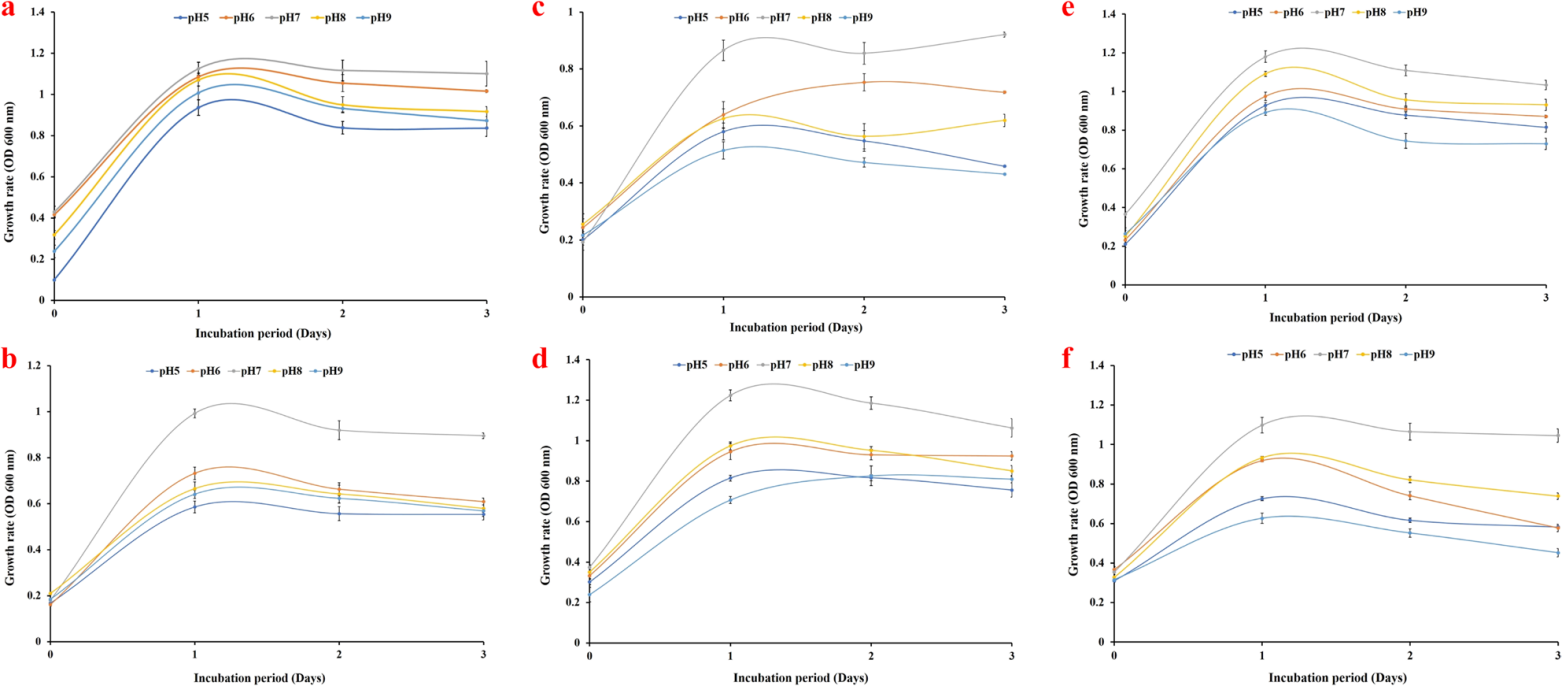
Effect of pH values on the growth rate of the isolates (**a**, **d**) B8-1, (**b**, **e**) A1, and (**c**, **f**) A3 on phenanthrene and naphthalene, respectively at 37 °C and 150 rpm. The values are presented as mean values ± SD, *n* = 3

**Fig. 4:**
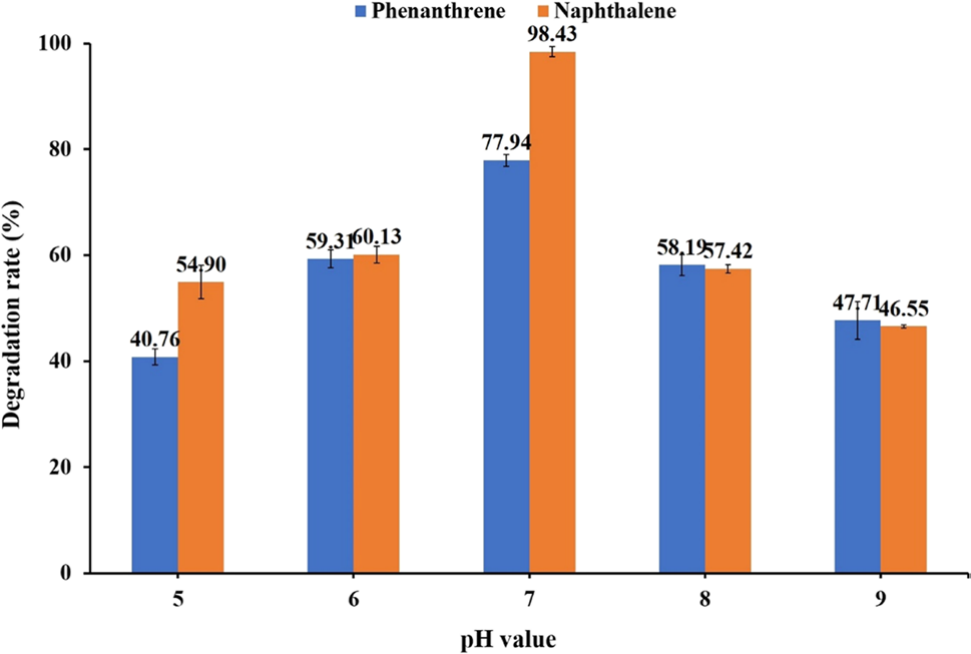
Effect of pH values on the phenanthrene and naphthalene degradation by the isolates B8-1 at 37 °C and 150 rpm after 7 days of incubation

The growth of the selected bacterial isolates on phenanthrene and naphthalene was evaluated at different pH values (5–9). As shown in (Fig. 3, a–f), all isolates were able to grow and utilize both compounds across the tested pH range, although the maximum growth was observed at pH 7. Among the tested isolates, B8-1 exhibited the highest growth rate on both phenanthrene and naphthalene. Therefore, isolate B8-1 was selected to further evaluate the effect of pH on PAH degradation by determining the residual concentrations of phenanthrene and naphthalene using HPLC. The degradation results showed that phenanthrene degradation exceeded 50% within the pH range of 6–8, with the maximum degradation rate (77.94%) observed at pH 7 (Fig. 4). Similarly, naphthalene degradation exceeded 55% between pH 5–8, reaching a maximum degradation rate of 98.43% at pH 7 (Fig. 4). These findings indicate that isolate B8-1 is capable of degrading PAHs across a relatively wide pH range. The preference for pH 7 may be attributed to the fact that the isolates were initially enriched and maintained in culture media with a neutral pH, which may have facilitated their physiological adaptation to this condition. Similar observations have been reported in previous studies, where the optimal pH for PAH biodegradation varied depending on the bacterial strain, environmental origin, and the specific hydrocarbon being degraded [31, 32].

#### Effect of temperature

**Fig. 5:**
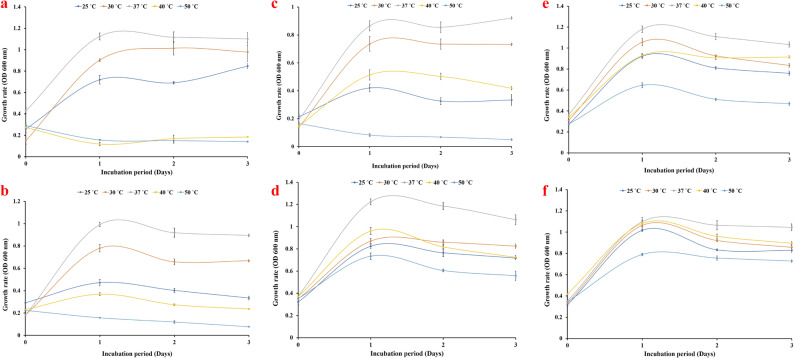
Effect of temperature on the growth rate of the isolates (**a**, **d**) B8-1, (**b**, **e**) A1, and (**c**, **f**) A3 on phenanthrene and naphthalene, respectively at pH 7 and 150 rpm. The values are presented as mean values ± SD, *n* = 3

**Fig. 6:**
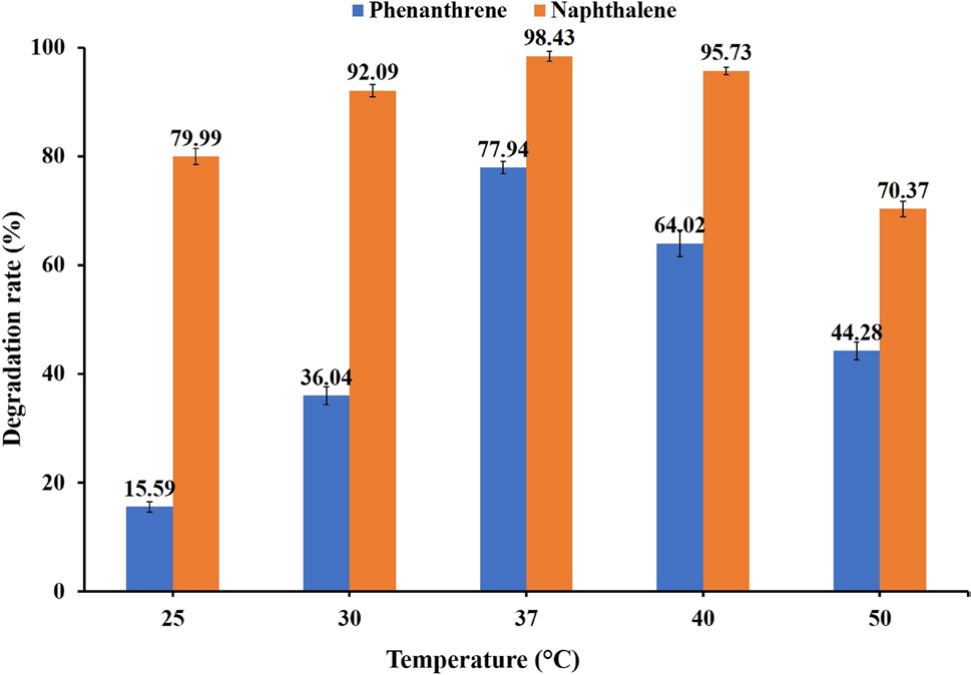
Effect of temperature on the phenanthrene and naphthalene degradation by the isolate B8-1 at pH 7 and 150 rpm after 7 days of incubation

The three isolates were subjected to different temperature ranges of 25, 30, 37, 40, and 50 °C, at a pH value of 7. (Figure 5 a-f) indicated that the three isolates were able to grow at different temperature ranges of 25–50 °C. The maximum growth rate was observed at 37 °C for the three selected isolates. The maximum growth rate was attained by the isolate B8-1. This result may be attributed to the mesophilic isolation site; therefore, its enzymes are adapted to these moderate temperatures. Similarly, as in the previous experiment, the effect of the same ranges of temperature on the biodegradation rate of phenanthrene and naphthalene by the isolate B8-1 was investigated. (Figure 6) demonstrated that the biodegradation rate of phenanthrene was constantly increased and then decreased over the experimental temperature ranges. The optimal temperature for phenanthrene biodegradation of 77.94% was achieved at 37^*◦*^C, then declined when the temperature reached 40 °C and above. In the case of naphthalene, isolate B8-1 showed a great biodegradation rate of 92.09%, 98.43%, and 95.73% of naphthalene at temperatures of 30, 37, and 40 °C, respectively (see Fig. 6). Moreover, at a high temperature of 50 °C, the isolate B8-1 has displayed a naphthalene biodegradation rate of 71.36%. This result indicated that this isolate can be efficient as a naphthalene bio-degrader under a wide range of temperatures. The isolate B8-1 has demonstrated the maximum biodegradation rate of 98.43% at 37 °C. These results suggested that temperature values influence on the bacterial growth rate and metabolic activity positively, as it increases PAHs solubility and accordingly bioavailability, which may enhance microbial PAHs biodegradation [33, 34]. However, at higher temperatures, the growth of the bacteria is reduced, and therefore, the degradation rate might be attributed to the negative effect of high temperature on the enzymatic activity, which controls the bacterial growth [35, 36].

### Induction, screening, and quantification of biofilm formation ability

**Fig. 7:**
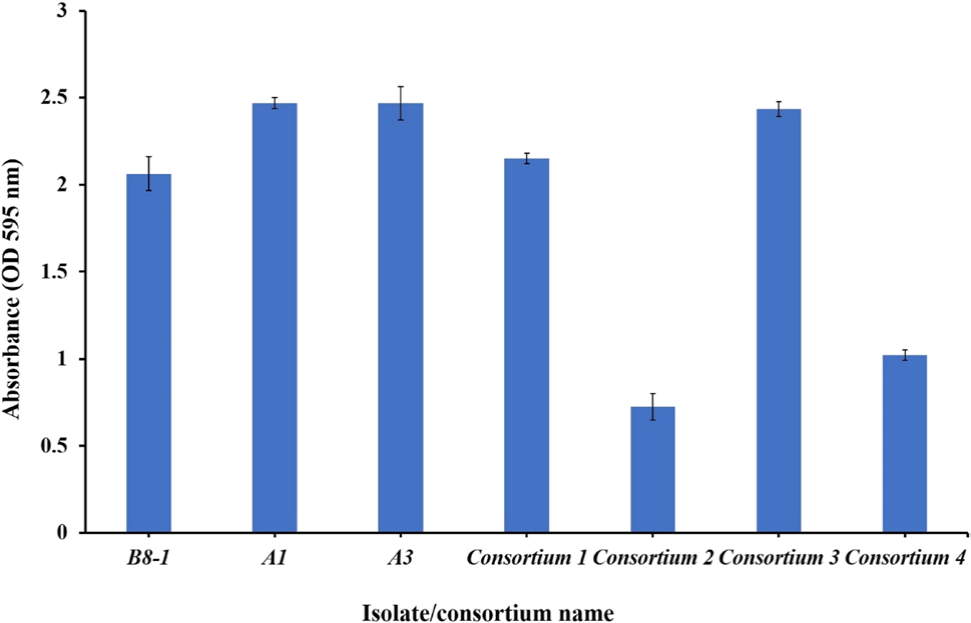
Quantitative determination of biofilm formation by crystal violet assay at an OD of 595 nm

The three selected isolates and the developed consortia were screened for biofilm formation ability by visualization of the attached cell-mass to the glass tube visible after crystal violet staining. Both the individual isolates and the consortia were able to form biofilm on the glass surface. The isolates with OD_595_ = 0.2 or more were reported as having a high ability to form biofilm and OD_595_ less than 0.2 were reported as having a poor ability to form biofilm, as previously reported [36], the results were displayed in Fig. 7.

### Comparison between the biodegradation rate of individual isolates and consortia in planktonic and biofilm growth modes

#### Phenanthrene biodegradation

**Fig. 8:**
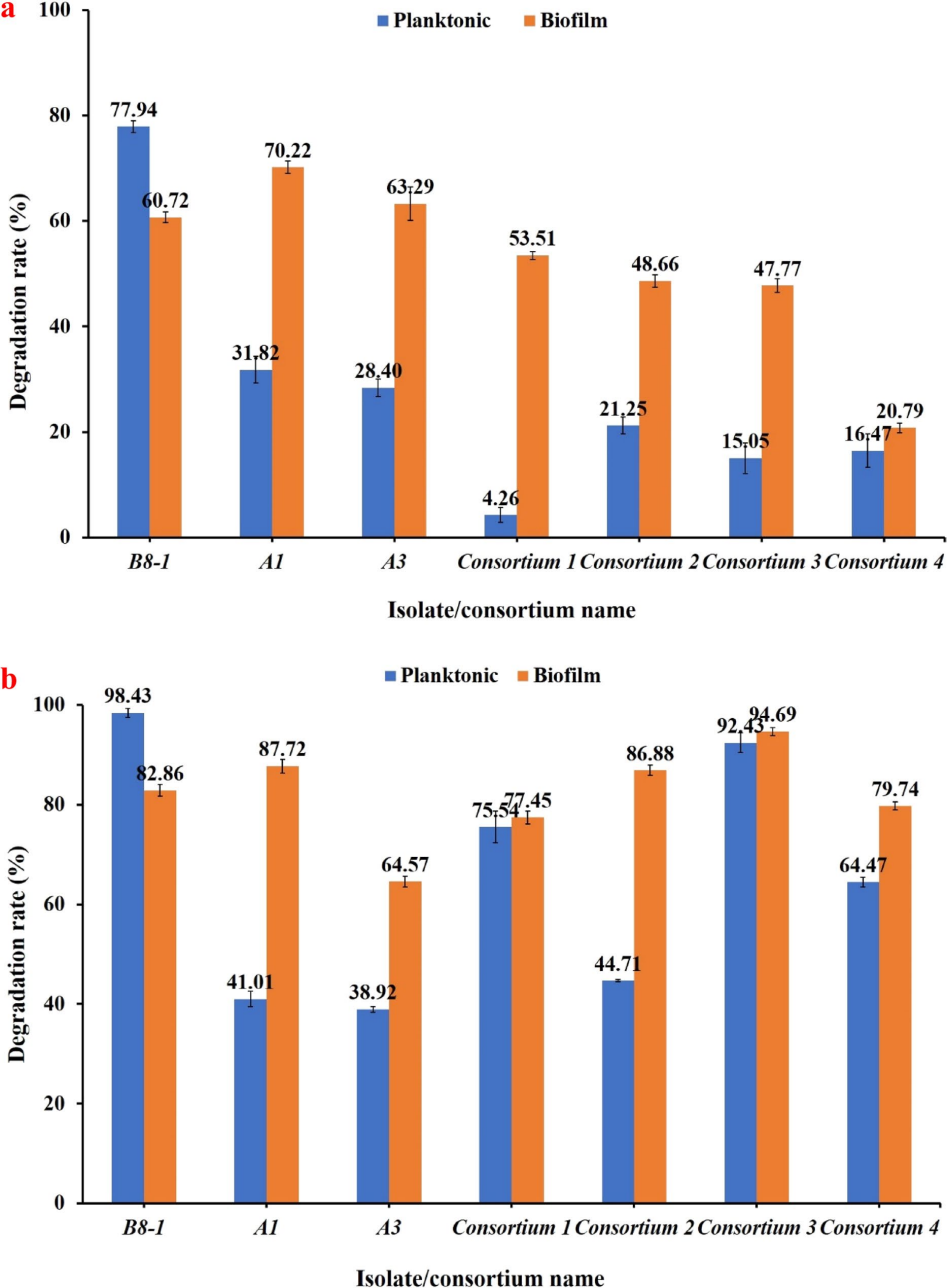
Comparison between degradation abilities by individual isolates and consortia in planktonic (**a**) and biofilm modes (**b**) on growth of phenanthrene and naphthalene

Phenanthrene biodegradation was studied under both planktonic and biofilm modes of growth using individual isolates and developed consortia at an initial concentration of 200 mg/l using HPLC. Figure 8a showed that the biofilm growth mode displayed about > 50% biodegradation rate by the isolates A1, A3, and B8-1. and consortium 1 biofilm. In addition, planktonic cultures of A1, A3, consortium 1, consortium 2, consortium 3, and consortium 4 displayed degradation rates of 31.82, 28.40, 4.26, 21.25, 15.05, and 16.47%, respectively. Whereas in the biofilm mode, the biodegradation rates were 70.22, 63.29, 53.51, 48.66, 47.77, and 20.79%, respectively. That means the biodegradation rate of phenanthrene was higher in the biofilm mode in comparison to its planktonic cell counterpart. This result suggested that, although the phenanthrene biodegradation rate remains a challenge for bioremediation due to its low solubility, the biofilm mode of growth was able to degrade it more efficiently when compared to planktonic cells. These findings agree with the results reported previously [37, 38], as they demonstrated that biofilm mode (78.7–85.5%) facilitated the biodegradation of phenanthrene more than planktonic cells (11–20%). This might be due to that the produced extracellular polymeric substances (EPS) of bacterial biofilms are associated with several surface-active functional groups, which enhance the bioavailability of PAHs, enhancing their solubility and then leads to enhanced biodegradation rate [9, 39]. Thus, these findings put forward the effectiveness of bacterial biofilms over planktonic culture in developing bioremediation technology of organic pollutants. On the contrary, the isolate B8-1 exhibited a biodegradation rate, in the planktonic mode, of 77.94% that relatively higher than that obtained in its biofilm mode, 60.72% (see Fig. 8a). Similar results were also observed previously [36]. This attempt may be attributed to the delayed time that phenanthrene takes to enter through the EPS matrix of this isolate in order to induce the genes responsible for phenanthrene degradation [40]. Surprisingly, the biodegradation rates by the individual isolates were higher than those of the consortium. This observation may relate to an antagonistic effect of the consortium isolates [41]. Moreover, these findings could be attributed to the nutrient stress and competition between the individual isolates, which inhibit the growth and metabolism of each other, thus affecting the outcome of bioremediation negatively, which is better described as an antagonistic effect [42]. Furthermore, the results showed that single strain B8-1 has the highest phenanthrene degrading capacity compared to microbial consortia with a 77.94% biodegradation rate. These results confirmed that the single strains B8-1, A1, and A3 showed a great potential in phenanthrene biodegradation as compared to developed consortia, which is similar to the results observed earlier [41, 43].

#### Naphthalene biodegradation

The isolates A1, A3, and B8-1 and their consortia showed some similarity to the phenanthrene in terms of biodegradation ability when naphthalene was supplemented to the culture medium (see Fig. 8b). The result demonstrated that the naphthalene biodegradation rates of 41.01, 38.92, 75.54, 44.71, 92.43, and 64.47% for the planktonic cultures, whereas were 87.72, 64.57, 77.45, 86.88, 94.69, and 79.74% for the biofilm cultures of isolates A1, A3, consortium 1, consortium 2, consortium 3, and consortium 4, respectively. Moreover, the result displayed that the biofilm cultures of isolates A1 and consortium 2 showed an increase in the naphthalene biodegradation rates from 41.01 to 87.72% and from 44.71 to 86.88%, respectively. This result means that the biofilm multiplied the naphthalene biodegradation rates by more than 2-fold when compared to the planktonic cells. This proves that the biofilm was more effective than the planktonic cells in naphthalene biodegrading. These results are in agreement with McGenity and his co-workers [42] ,who reported that the naphthalene degradation by *Pseudomonas aeruginosa* N6P6 biofilm and planktonic culture was 99.4 ± 0.002% and 77 ± 3.25%, respectively. However, as occurred with phenanthrene, the degradation rate of naphthalene by the planktonic culture of B8-1 was higher than that of its biofilm culture. Moreover, the results exhibited that the biofilms of consortia 2 and 3 have higher biodegradation ability than the biofilm cultures of all individual isolates. Moreover, the planktonic cultures of all consortia have more naphthalene biodegradation ability than the planktonic cultures of A1 and A3 individual isolates. This finding demonstrated the synergistic interaction between the consortia`s members, with the highest synergistic interaction between the planktonic isolates (A1 and A3) in the consortium 3 and the least synergistic interaction between the planktonic isolates (B8-1 and A3) in the consortium 2. One possible reason that makes the bacterial consortia have a superior naphthalene biodegradation efficiency is that, during the process of biotransformation, the product of one bacterium can be employed as the foundation for the catabolism and growth of another. In addition, this finding results are similar to the results observed by Li and his co-workers [44], who reported that a mixed bacterial community attained about 99% biodegradation rate After 5 days. Li and his team [45] also reported an improved in the biodegradation ability by microbial consortia than single strains, which may be attributed to synergistic interactions between members of the consortium.

### Phenanthrene and naphthalene mixture biodegradation using planktonic cultures and biofilm cultures

**Fig. 9:**
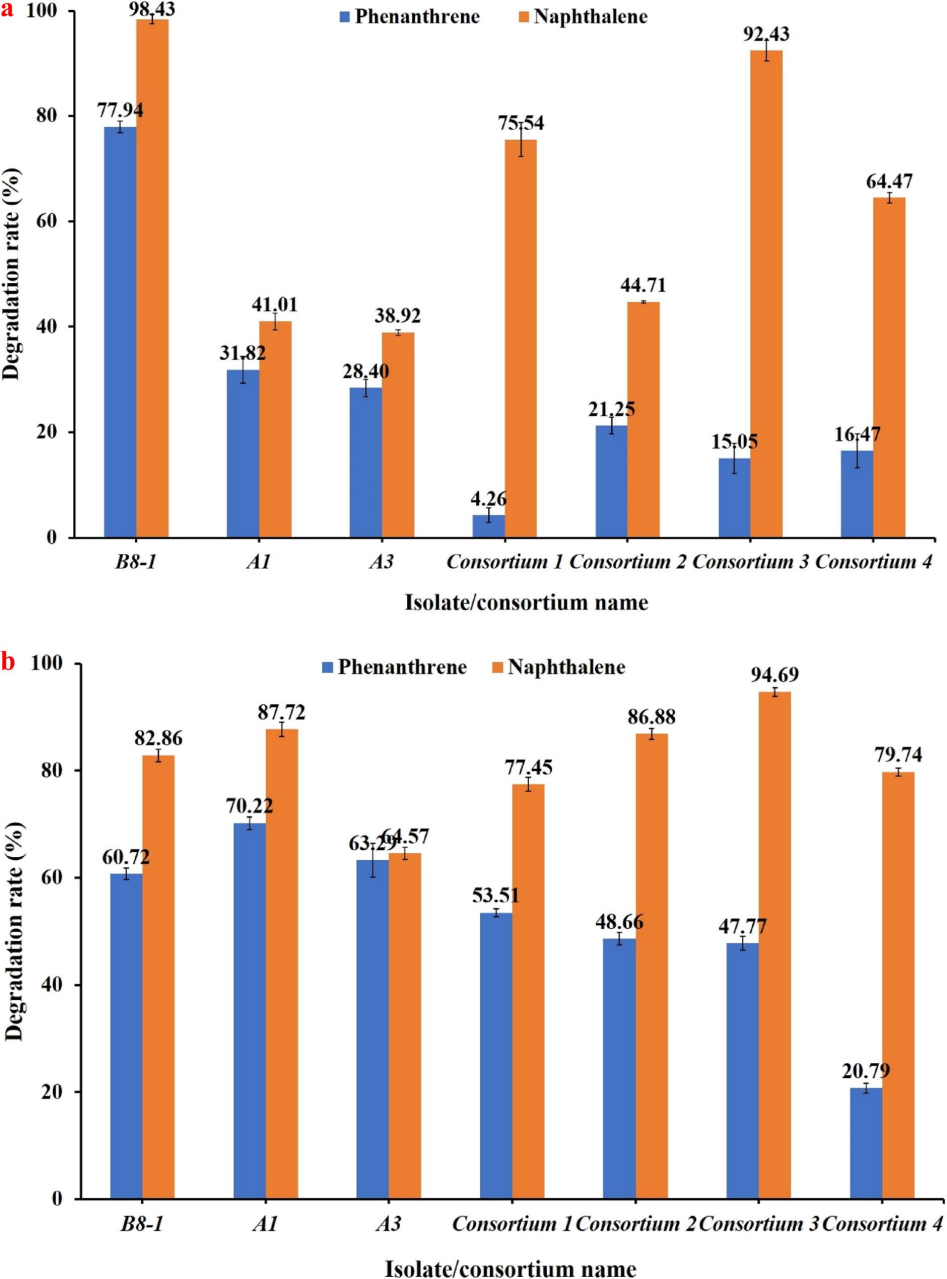
Comparison between phenanthrene and naphthalene mixture biodegradation using (**a**) planktonic cultures (**b**) and biofilm cultures

Naphthalene is more biodegradable than phenanthrene by isolates and their consortia (Fig. 9a). The reason may be attributed to the fact that naphthalene is simpler in its structure than phenanthrene. Hence, naphthalene is more soluble and bioavailable to be degraded by the bacterial isolates. In a study conducted by Ni’matuzahroh et al., 2017 [46], higher biodegradation of naphthalene was attained compared to phenanthrene, with a degradation rate of 70.5 and 24.8%, respectively. Parab et al., 2020 [47] also reported that consortia of (*Chryseobacterium* sp., *Sphingobacterium* sp., *Stenotrophomonas* sp., *Agromyces* sp., and *Pseudomonas* sp.) were able to degrade 99.9% of naphthalene and 92.9% of phenanthrene as a mixture at a concentration of 2000 mg/l. Moreover, our results demonstrated that the simple structure of naphthalene induced the synergistic interaction between members of the consortia. However, in the case of phenanthrene, there is an antagonistic interaction between members of the consortia.

The isolates A1 and A3 in the consortium 3, and the isolates B8-1 and A3 in the consortium 2, showed a synergistic effect in the case of naphthalene (Fig. 9b). However, in the case of phenanthrene, there is an antagonistic effect between biofilm consortia members. This finding implies that this behaviour of the interaction between bacteria is influenced by the substrate’s chemical complexity. This is shown here in naphthalene, a less complex substrate, that promotes positive interactions and synergistic growth. In the case of phenanthrene, a complex substrate, it promotes antagonistic interactions and negative growth [47]. The higher degradation efficiency observed for naphthalene compared with phenanthrene may be attributed to differences in their metabolic pathways. Naphthalene degradation generally proceeds through relatively simple enzymatic pathways involving naphthalene dioxygenase and subsequent conversion to salicylate and catechol intermediates [47]. In contrast, phenanthrene degradation requires multiple enzymatic steps and a broader range of enzymes, including ring-hydroxylating dioxygenases and ring-cleavage dioxygenases, to transform complex aromatic structures into metabolizable intermediates [48]. This higher metabolic complexity, phenanthrene degradation may require cooperative interactions among microbial populations, which can influence degradation efficiency in microbial consortia [49].

### Evaluating the efficiency of biofilm cells in the biodegradation of PAHs in artificially contaminated soils

**Fig. 10:**
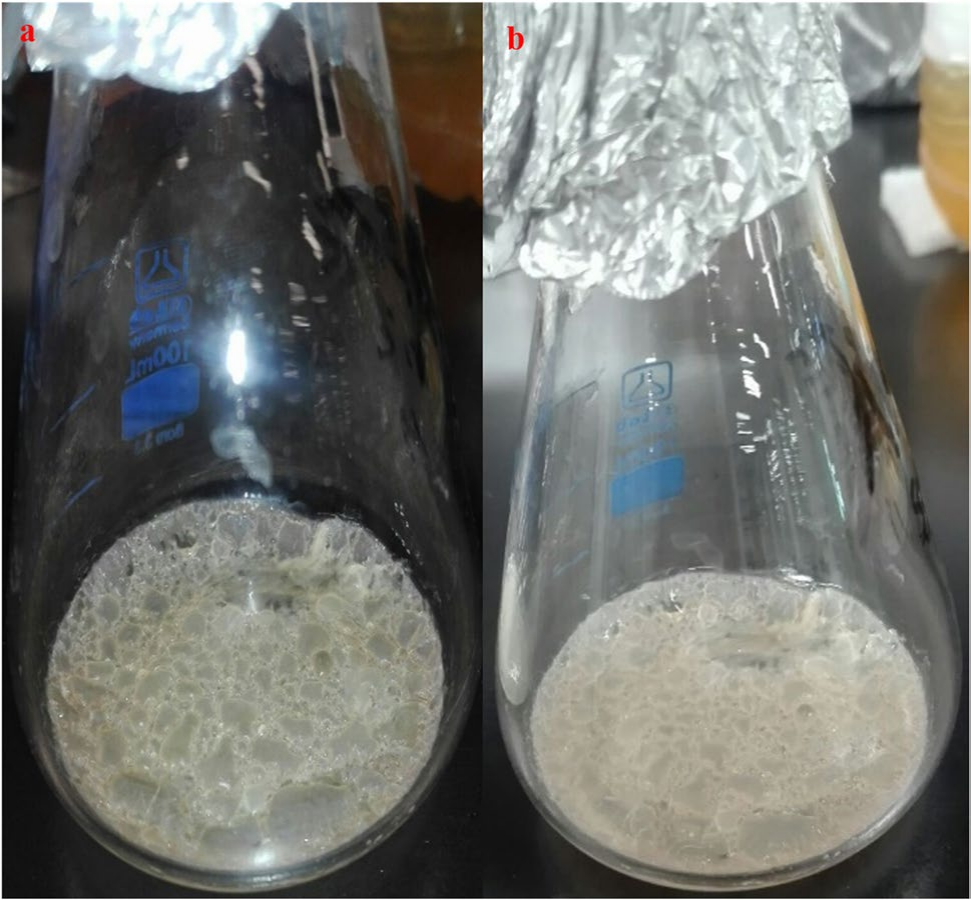
Layer of biofilm cells attached to the wall of the flasks of the isolate (**a**) A1 and (**b**) consortium 3

**Fig. 11:**
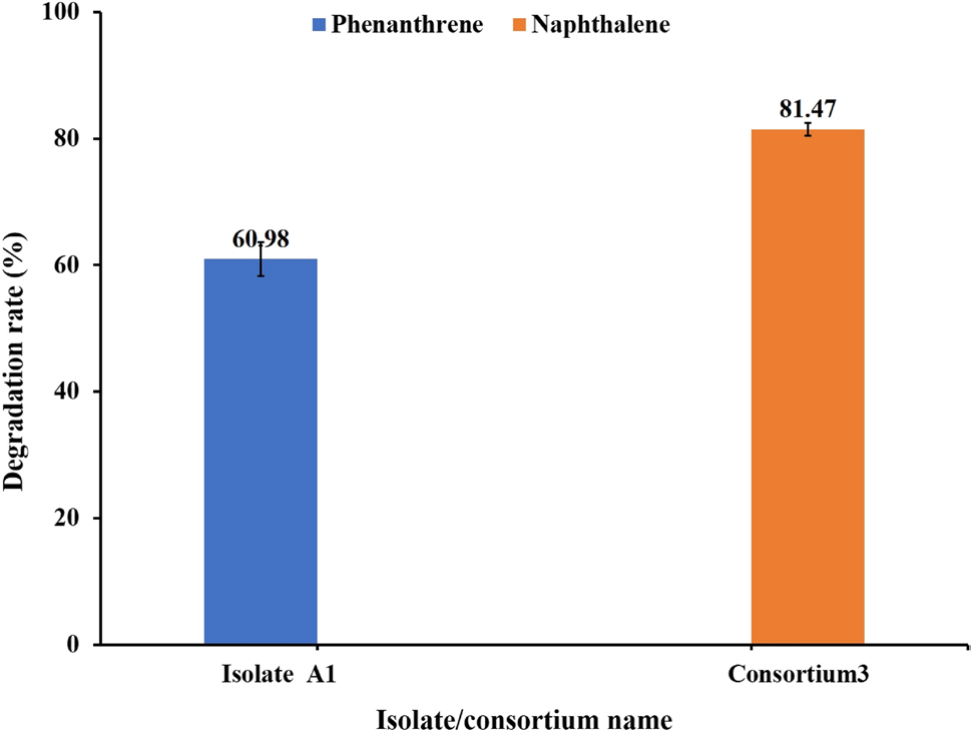
Degradation percentage of biofilm cells of isolate A1 and consortium 3 in in artificially contaminated soils

Biofilms of the isolate A1 and the consortium 3, which showed the maximum biodegradation rates among other isolates on phenanthrene and naphthalene, respectively, were formed on the walls of the flasks, as shown in Fig. 10. The HPLC results exhibited that the biofilm of isolate A1 degraded 60.98% of phenanthrene, and the biofilm of the consortium 3 degraded 81.47% of naphthalene in soil samples, as shown in Fig. 11. This attempt recommends the application of biofilm in PAH degradation field experiments.

### Molecular identification and phylogenetic studies

**Fig. 12:**
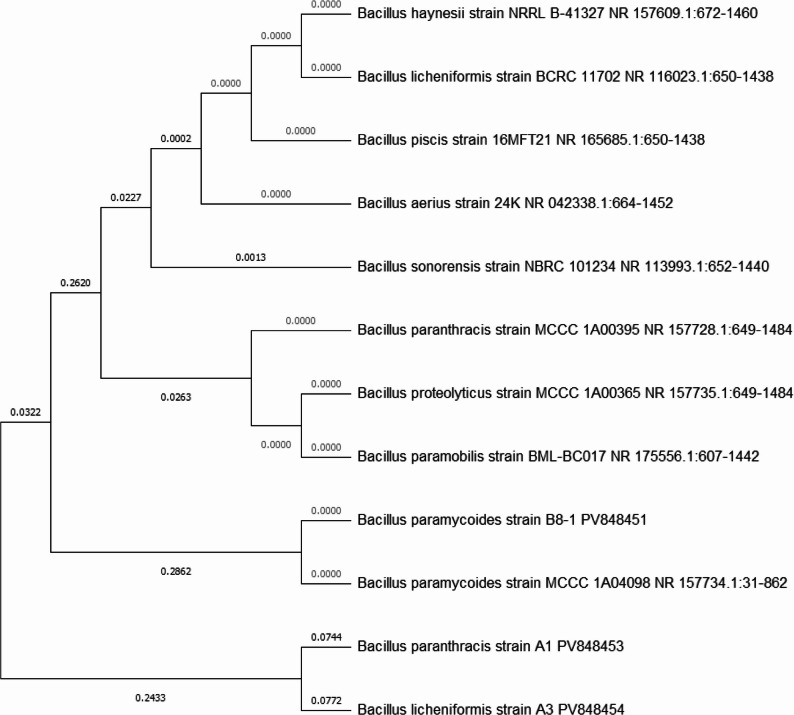
Phylogenetic tree of 16 S rRNA gene sequence of the isolate B8-1 and the nearest relatives retrieved from NCBI database

The three isolates were selected for molecular identification as the most potent PAH-degrading bacteria. A phylogenetic tree (see Fig. 12) was constructed using Mega software using the neighbour-joining method on the obtained 16 S rRNA coding gene sequences of the isolates and the nearest relatives’ isolates retrieved from the database of the National Centre for Biotechnology Information (NCBI). The phylogenetic sequence analysis of the three isolates was affiliated as B8-1(*Bacillus paramycoides*), A1(*B Bacillus paranthracis*), and A3(*Bacillus licheniformis*).

## Conclusion

In this study, efficient polycyclic aromatic hydrocarbon (PAH)-degrading bacteria were successfully isolated and characterized. The results demonstrated that the optimization of environmental parameters significantly enhanced the biodegradation of PAHs. The isolates showed maximum degradation efficiency at pH 7.0 and 37 °C with a PAH concentration of 200 mg/L. Among the isolates, *Bacillus paramycoides* exhibited the highest degradation ability, degrading 77.94% of phenanthrene and 98.43% of naphthalene.

Furthermore, the isolated strains were used to develop bacterial consortia and biofilms. Comparative experiments between planktonic and biofilm cultures revealed that the biofilm mode of growth significantly enhanced PAH degradation compared to planktonic cells. The degradation studies also showed that substrate complexity influences bacterial interactions within consortia. Complex substrates such as phenanthrene promoted antagonistic interactions, resulting in lower degradation efficiency, whereas simpler substrates such as naphthalene promoted synergistic interactions and enhanced biodegradation.

Finally, the biofilms of *Bacillus paranthracis* and consortium 3, which showed the highest degradation capacities for phenanthrene and naphthalene, respectively, were tested in artificially contaminated soil. The results demonstrated that the biofilm of *Bacillus paranthracis* degraded 60.98% of phenanthrene, while the biofilm of consortium 3 degraded 81.47% of naphthalene. These findings highlight the potential application of these bacterial isolates and their biofilms as effective agents for the bioremediation of PAH-contaminated environments. 

## Data Availability

The DNA sequences of the used bacterial isolates during the current study were deposited to the NCBI Genbank, and available in the NCBI database under accession numbers (PV848451, PV848453, and PV848454).

## Abbreviations

PAH: Polycyclic Aromatic Hydrocarbons
DCPIP: 2,6-Dichlorophenol Indophenol
MSM: Mineral Salt Medium
PBS: Phosphate Buffer Saline
PCR: Polymerase Chain Reaction
LB: Luria Broth
OD: Optical Density
HPLC: High-Performance Liquid Chromatography
rpm: Revolutions per Minute
NCBI: National Center for Biotechnology Information
EPA: Environmental Protection Agency
TSB: Trypticase Soy Broth
DMSO: Dimethyl sulfoxide
CFU: Colony Forming Units
BLAST: Basic Local Alignment Search Tool
